# A Retrospective Review of 30-Day Hospital Readmission Risk After Open Heart Surgery in Patients With Atrial Fibrillation

**DOI:** 10.7759/cureus.45755

**Published:** 2023-09-22

**Authors:** Varun Rao, Genaro DeLeon, Aish Thamba, Mindy Flanagan, Kathleen Nickel, Michael Gerue, Douglas Gray

**Affiliations:** 1 Department of Neurological Surgery, Indiana University School of Medicine, Indianapolis, USA; 2 Department of General Surgery, Indiana University School of Medicine, Indianapolis, USA; 3 Department of Research and Innovation, Parkview Mirro Center for Research and Innovation, Parkview Health, Fort Wayne, USA; 4 Department of Cardiovascular Surgery, Parkview Heart Institute, Parkview Health, Fort Wayne, USA

**Keywords:** atrial fibrillation (afib), patient readmission, postoperative complications, telemedicine services, open heart surgery (ohs)

## Abstract

Introduction

Readmission rates after open heart surgery (OHS) remain an important clinical issue. The causes are varied, with identifying risk factors potentially providing valuable information to reduce healthcare costs and the rate of post-operative complications. This study aimed to characterize the reasons for 30-day hospital readmission rates of patients after open heart surgery.

Methods

All patients over 18 years of age undergoing OHS at a community hospital from January 2020 through December 2020 were identified. Demographic data, medical history, operative reports, post-operative complications, and telehealth interventions were obtained through chart review. Descriptive statistics and readmission rates were calculated, along with a logistic regression model, to understand the effects of medical history on readmission.

Results

A total of 357 OHS patients met the inclusion criteria for the study. Within the population, 8.68% of patients experienced readmission, 10.08% had an emergency department (ED) visit, and 95.80% had an outpatient office visit. A history of atrial fibrillation (AFib) significantly predicted 30-day hospital readmissions but not ED or outpatient office visits. Telehealth education was delivered to 66.11% of patients.

Conclusion

The study investigated factors associated with 30-day readmission following OHS. AFib patients were more likely to be readmitted than patients without atrial fibrillation. No other predictors of readmission, ED visits, or outpatient office visits were found. Patients reporting symptoms of tachycardia, pain, dyspnea, or "other" could be at increased risk for readmission.

## Introduction

Recent studies have demonstrated high readmission rates after heart valve surgery and coronary artery bypass graft surgery (CABG) [1-7]. Readmissions affect the quality of life, raise healthcare costs, and increase the risk of hospital-acquired complications [8,9]. The causes of readmission after heart valve surgery are variable, including chest pain, heart failure, arrhythmia, wound complications, pericardial effusions, and pleural effusions [1-4,10]. Therefore, post-discharge is crucial to symptom identification, as patients are vulnerable to adverse events, lapses in care, and readmission [8,11].

The coronavirus disease 2019 (COVID-19) pandemic has seen a national shift toward telehealth services [12]. The relevance of telemedicine in post-operative care has become increasingly important due to the COVID-19 pandemic, causing hospitals and their cardiac surgery programs to transition to distance models using telehealth platforms, intending to minimize the risk of COVID-19 infection among patients and their providers [13]. Despite the increased adoption of telemedicine and recent demand during the pandemic, few studies have examined its effectiveness in cardiac surgery patients. The study aimed to describe the causes of 30-day hospital readmission, ED visits, and outpatient visits of patients receiving non-telehealth and telehealth post-operative care after open heart surgery (OHS).

## Materials and methods

Informed patient consent was not applicable since this study is a retrospective cohort study involving retrospective chart review. The data is anonymized, cannot be linked to specific individuals, and is exempted from the Parkview Health Research Ethics Committee's Institutional Review Board approval by falling into the exempt human subject research category.

All adult (≥18 years) patients undergoing OHS at Parkview Heart Institute from January 2020 through December 2020 were included in the study. Electronic medical records were reviewed to collect patient demographics, medical history, operative reports, and post-operative documents. Patient demographic information, such as age, gender, race, ethnicity, and relevant medical history data were collected. Relevant medical history included the following variables: atrial fibrillation (AFib), previous myocardial infarction within 30 days of surgery, congestive heart failure (CHF), stroke, diabetes, pre-operative glycated hemoglobin (HbA1C), asthma, chronic obstructive pulmonary disorder (COPD), and hemodialysis. Operative reports were used to determine surgery type, surgery length, and intra-operative complications. Post-operative records were reviewed to identify complications, such as AFib, pleural effusion, sternal wound complications (SWC), shortness of breath (SOB), fluid overload, and infection. The Philips eCareCoordinator platform was reviewed to collect telehealth intervention data reported by patients as part of the Parkview Health Telehealth program.

Descriptive statistics and readmission rates were calculated. History of myocardial infarction (within 30 days), CHF, stroke, diabetes, asthma, and COPD were investigated for effects on 30-day readmission after OHS in a logistic model. Patients post-operatively were defined as either undergoing observation stays, readmissions, or emergency department (ED) visits. Observation stays were defined as patients who were temporarily admitted inpatient for less than 24 hours by hospital outpatient services to decide whether an inpatient admission was necessary. Readmissions were defined as patients who had been discharged recently and readmitted to the hospital for the same or related care within 30, 60, or 90 days of discharge. ED visits were defined as patients who sought care and received personal health services in the affiliated emergency department. Models included age, surgery type (coronary artery bypass graft {CABG} only, valve only, CABG/valve combined, or other), discharge status (home vs. home health), and surgery length as covariates. Additionally, a logistic regression model was tested to analyze the impact of medical history on 30-day ED visits. Readmission groups were compared on each symptom (yes/no) for the patients who reported symptoms during telehealth visits. Due to the large number of comparisons, p-values were adjusted using the false discovery rate approach [14].

## Results

A total of 357 patients met the inclusion criteria for this study. Only 8.68% (31/357) of patients experienced hospital readmission, 10.08% (36/357) had an ED visit, 95.80% (342/357) had at least one outpatient visit, and 2.52% (9/357) had an observation stay. Patients with a history of AFib comprised 24.65% (88/357) of the sample. Complete sample characteristics are shown in Table 1.

**Table 1:** The characteristics of the patient population involved in the study.

Characteristics		Full sample (n=357)	Without readmission (n=326)	With readmission (n=31)	Difference test statistic, p-Value
Age (years), mean (SD)		65.81 (11.30)	65.77 (11.26)	66.23 (11.88)	t (355)=0.22, p=0.83
Gender, n (%)	Female	97 (27.17)	88 (26.99)	9 (29.0328)	χ^2^(1)=0.06, p=0.81
	Male	260 (72.83)	238 (73.01)	22 (70.97)	
Race, n (%)	Black	9 (2.52)	7 (2.15)	2 (6.45)	p=0.38
	White	345 (96.64)	316 (96.93)	29 (93.55)	
	Other	3 (0.84)	3 (0.92)	0 (0.00)	
Ethnicity, n (%)	Hispanic	7 (1.96)	6 (1.84)	1 (3.23)	p=0.47
	Not Hispanic	350 (98.04)	320 (98.16)	30 (96.77)	
Medical history, n (%)	Atrial fibrillation (AFib)	88 (24.65)	75 (23.01)	13 (41.94)	χ^2^(1)=5.46, p=0.02
	Myocardial infarction (MI) (within 30 days)	64 (17.93)	59 (18.10)	5 (16.13)	χ^2^(1)=0.07, p=0.78
	Congestive heart failure (CHF)	25 (7.00)	22 (6.75)	3 (9.68)	p=0.47
	Stroke	22 (6.16)	19 (5.83)	3 (9.68)	p=0.42
	Diabetes	122 (34.17)	109 (33.44)	13 (41.94)	χ^2^(1)=0.91, p=0.34
	Asthma	14 (3.92)	13 (3.99)	1 (3.23)	p<0.99
	Chronic obstructive pulmonary disorder (COPD)	40 (11.20)	37 (11.35)	3 (9.68)	p<0.99
	Hemodialysis	3 (0.84)	3 (0.92)	0 (0.00)	p<0.99
Surgery type, n (%)	Coronary artery bypass graft (CABG) only	184 (51.69)	166 (51.08)	18 (58.06)	p=0.78
	Valve only	127 (35.67)	118 (36.31)	9 (29.03)	
	Combined valve/CABG	18 (5.06)	17 (5.23)	1 (3.23)	
	Other	27 (7.58)	24 (7.38)	3 (9.68)	
Surgical complications, n (%)	Yes	6 (1.69)	5 (1.54)	1 (3.23)	p=0.42
	No	350 (98.31)	320 (98.46)	30 (96.77)	
Surgery length (minutes), mean (SD)		274.45 (118.64)	273.10 (117.00)	289.10 (136.20)	t (355)=0.72, p=0.47
Discharge status, n (%)	Home	238 (66.67)	218 (66.87)	20 (64.52)	χ^2^(1)=0.07, p=0.79
	Home healthcare	119 (33.33)	108 (33.13)	11 (35.48)	

As shown in Table 2, the most frequent reason for readmission was "other" and the second most frequent reasons were AFib and SOB. For patients with a history of AFib and readmission, the most frequent reasons for admission were "other" (n=5), AFib (n=3), and pleural effusion (n=2).

**Table 2:** Frequency percentage of reasons for readmission, ED visit, and outpatient visit. Presentation of the percentages of readmission, emergency department visits, and outpatient visits in the patient population studied.

Reason	Readmission (n=31)	ED (n=36)	Outpatient visit (n=342)
Atrial fibrillation (AFib)	7 (22.58)	4 (11.11)	40 (11.70)
Pleural effusion	4 (12.90)	2 (5.56)	7 (2.05)
Sternal wound complication	0 (0.00)	1 (2.78)	17 (4.97)
Shortness of breath (SOB)	7 (22.58)	5 (13.89)	46 (13.45)
Syncope	1 (3.23)	3 (8.33)	2 (0.58)
Claudication	0 (0.00)	0 (0.00)	2 (0.58)
Dehydration	1 (3.23)	0 (0.00)	1 (0.29)
Infection	3 (9.68)	1 (2.78)	9 (2.63)
Tachycardia	0 (0.00)	2 (5.56)	5 (1.46)
Fluid overload	6 (19.35)	2 (5.56)	23 (6.73)
Pulmonary embolism	0 (0.00)	0 (0.00)	0 (0.00)
Other	17 (54.84)	23 (63.89)	339 (99.12)

Telehealth education was delivered to a percentage of the sample, 66.11% (263/357). Of these, during telehealth visits, 98.48% (259/263) received post-operative care education, 49.05% (129/263) reported symptoms, and 4.94% (13/263) made an office visit for a follow-up appointment after the telehealth encounter. The selection of patients for symptom reporting was significantly related to a medical history of AFib, χ^2^(1)=5.93, p=0.01. For patients with AFib, 61.8% (42/68) were asked to report symptoms; in comparison, for patients without AFib, 44.6% (87/195) reported symptoms.

For telehealth patients reporting symptoms, those with readmission were more likely to report tachycardia and "other" symptoms with a trend toward significance with respect to adjusted p-values than corresponding patients without readmission (Table 3).

**Table 3:** Presentation of the percentages of call reasons for post-operative symptoms and readmission rates reported by patients utilizing telehealth services.

Symptom	Without readmission (n=112), %	With readmission (n=17), %	Adjusted p-Value
Hypertension	62.50	58.82	0.89
Hypotension	55.36	76.47	0.30
Tachycardia	32.14	64.71	0.07
Bradycardia	36.61	23.53	0.52
Low pulse oximetry reading	19.64	23.53	0.89
Sudden weight gain	47.32	58.82	0.57
Sudden weight loss	84.82	88.24	0.89
Temperature ≥100°F twice in 24 hours	5.36	11.76	0.52
New onset or uncontrolled pain	13.39	35.29	0.11
Feeling of heart racing or pounding	27.68	29.41	0.92
New onset or symptomatic dyspnea	26.79	52.94	0.11
New onset or worsening cough	11.61	23.53	0.38
New onset or increased swelling of legs	33.93	52.94	0.33
Changes in incision/wound appearance	36.61	35.29	0.92
Other	7.14	29.41	0.07

History of AFib was significantly related to readmission (adjusted odds ratio=2.72; 95% CI=1.20, 6.07) but not associated with ED visits or the number of outpatient visits (Table 4). The readmissions were related to the presence of Afib, not complications secondary to Afib. No other significant predictors of readmission were discovered.

**Table 4:** Logistic regression model predicting 30-day hospital readmission after surgery (n=357). Presentation of the summary of results utilizing the logistic regression model predicting 30-day post-operative hospital readmission.

Parameter	Estimate	Standard error	Chi-square	p-Value
Intercept	-3.21	1.61	3.98	0.046
Age	0.005	0.02	0.07	0.80
History of atrial fibrillation (AFib)	0.99	0.41	5.93	0.01
Combined valve/coronary artery bypass graft (CABG) or coronary artery bypass graft (CABG)	-0.84	1.08	0.60	0.44
Other vs. coronary artery bypass graft (CABG) only	-0.06	0.75	0.01	0.94
Valve only vs. coronary artery bypass graft (CABG) only	-0.44	0.51	0.77	0.38
Home vs. home health	0.16	0.42	0.15	0.70
Operative room length of time (minutes)	0.001	0.002	0.22	0.64

## Discussion

Our study demonstrated that a history of AFib is associated with increased readmission rates. No other significant predictors of readmission were found. Most frequently, patients were readmitted with diagnoses listed under "other," AFib, and SOB. Predictors of readmission and reasons for readmission were studied 30 days post-operatively. Our study found no significant predictors for post-operative ED or outpatient office visits.

A history of AFib was the only predictor of readmission in our study. Readmission rates were significantly lower in patients without AFib. Other studies investigating predictors of readmission found AFib as one of the highest predisposing factors of readmission post-OHS [15-19]. Given these findings, our study suggests that greater care regarding pre-operative management of AFib should be considered when performing OHS on these patients to reduce rates of readmission.

The most common causes of readmission in patients with a history of AFib included "other," AFib, and pleural effusions. Weiss et al. and Trooboff et al. reported the presence of pleural effusions as the predominant reason for readmission in OHS patients [20,21]. Our study indicates that pleural effusions are a significant reason for readmission in the general OHS patient population and the history of AFib patient population. This is supported by literature that reports the presence of pleural effusion as a significant reason for hospital readmission in post-OHS patients [20,21].

In terms of reasons for overall readmission, patients were most likely readmitted for "other," AFib, and SOB. This supports literature evidence, which concludes that the most common cause of readmission post-OHS was non-cardiac-related, as represented by our "other" categorical variable [22-24]. This variable includes infections, gastrointestinal complications, and neurological damage. Although AFib remains one of the most studied causes of readmission post-OHS, we demonstrate the need to consider non-cardiac-related complications as well [15-20]. It is also worthwhile to consider a more qualitative review of these "other" cases to examine any common themes or patterns potentially impacting the quality of life post-operatively, given the diversity of symptoms experienced by patients.

The second most common cause of readmission among our patient population was AFib. Post-operative complications have been associated with increased rates of stroke, renal failure, and infections, leading to patient readmission [25,26]. SOB showed similar rates of readmission compared with AFib. Generalized SOB post-OHS has yet to be considered as a reason for readmission in the literature, and thus, our study introduces another admission factor to consider. Reducing the rates of "other," AFib, and SOB post-operatively could reduce hospital costs and readmission rates.

The study included a subset of patients receiving telehealth monitored by on-call nursing staff. Patients with AFib were more likely asked to report symptoms to telehealth nurses than those without AFib. In the telehealth population, reported symptoms, such as tachycardia, new onset or uncontrolled pain, new onset of symptomatic dyspnea or symptomatic dyspnea, or "other" symptoms, could have increased the risk of readmission. These symptoms may have been related to AFib. Increased readmission rates among patients with a history of AFib may also be related to increased symptom monitoring by telehealth nurses. This may have improved patient outcomes and reduced hospital costs as the subset of patients with a history of AFib receiving telehealth may have been instructed to visit the hospital by telehealth nurses.

Several limitations of our study need to be considered. This retrospective chart review was conducted at a single site, so the study may not be applicable to other patient populations. Multi-site studies could further expand upon our findings in future research. As with any retrospective chart review, our study was also susceptible to confounding bias. Subsequently, the study lacked sufficient racial and gender diversity as most patients receiving OHS at Parkview Health in 2020 were White males. Per a pilot study at the center, patients were subdivided into telehealth and non-telehealth groups for post-operative care. This organization may have affected rates of readmissions, ED presentations, and outpatient office visits as the telehealth group was monitored and instructed by nursing staff. While our study adjusted for several confounding variables, the severity of the initial nidus of heart disease was not considered, which begets a potential direction of future research.

Furthermore, the study was limited to data gathered in the year 2020 during the COVID-19 pandemic, which may have impacted readmission rates through lower adherence to medication regimens and the ability to access healthcare due to socioeconomic status. Moreover, the increased confidence interval of 1.20 to 6.07 affiliated with the adjusted odds ratio for the history of atrial fibrillation variable with respect to readmission rates indicates uncertainty. Due to a smaller sample size of 31 out of 357 patients experiencing readmission, this further reduces statistical power to detect significant predictors of post-operative hospital readmissions. Lastly, complications post-OHS often occur in relation to one another and may be related to multiple pre-operative conditions, including AFib. 

Future areas of investigation include the application of telehealth programs in post-operative OHS care. Studies show both improved patient and provider satisfaction with telemedicine monitoring post-OHS [27,28]. Furthermore, post-cardiac surgery telemedicine programs were able to detect dangerous complications [29]. Our study shows similar results regarding complication detection, as evidenced by our high rate of education delivery and symptom reporting. While our study provided a pilot for this center’s telemedicine program, we believe the literature and our own experiences support the safety, continued use, and further investigation into telemedicine programs in post-operative OHS care. Implementing telemedicine programs in cardiac surgery populations may lead to reduced readmission rates and improved outcomes for those with a history of AFib with further evaluation and remodeling.

Another potential direction of research would be the evaluation of long-term outcomes and complications in quality of life post-OHS after the time period used in this study of 30 days. It would also be intriguing to consider not adjusting p-values for multiple comparisons to find more meaningful associations. While our study focused on understanding readmissions within the context of patients who underwent OHS, it would be interesting to compare OHS patient readmission rates to those of other patients who underwent non-OHS procedures. This would require the utilization of interaction term analyses or machine learning algorithms in an attempt to understand the influence of multi-morbidities on post-operative readmission risk.

Lastly, as our study focuses on the influence of AFib on post-OHS recovery, the exploration of the influence of optimizing pre-operative atrial fibrillation management strategies, which range from pharmacological agents to lifestyle interventions, on reducing readmission risks has potential. Given the inherently complex nature of patients undergoing OHS, the associations of all the aforementioned variables regarding readmission may be reduced. However, as shown in our study, AFib was a predictor of readmission.

## Conclusions

Our study investigated factors contributing to 30-day readmission after discharge for OHS. Patients with AFib were more likely to be readmitted than patients without AFib. Due to the small sample size, symptom reports did not reveal significant differences between readmission groups. However, the data analysis suggested that patients reporting tachycardia, new onset or uncontrolled pain, new onset or symptomatic dyspnea, or "other" symptoms could be at risk for readmission. Regarding telehealth in post-OHS patient monitoring, we believe the pilot model shows promise in improving the quality of post-operative patient care but requires further investigation.
